# Host population bottlenecks drive parasite extinction during antagonistic coevolution

**DOI:** 10.1111/evo.12837

**Published:** 2015-12-30

**Authors:** Elze Hesse, Angus Buckling

**Affiliations:** ^1^ ESI, BiosciencesUniversity of ExeterPenryn CampusPenrynTR10 9FEUnited Kingdom

**Keywords:** Coevolutionary asymmetry, genetic drift, host diversity, local adaptation, population size, stochasticity

## Abstract

Host–parasite interactions are often characterized by large fluctuations in host population size, and we investigated how such host bottlenecks affected coevolution between a bacterium and a virus. Previous theory suggests that host bottlenecks should provide parasites with an evolutionary advantage, but instead we found that phages were rapidly driven to extinction when coevolving with hosts exposed to large genetic bottlenecks. This was caused by the stochastic loss of sensitive bacteria, which are required for phage persistence and infectivity evolution. Our findings emphasize the importance of feedbacks between ecological and coevolutionary dynamics, and how this feedback can qualitatively alter coevolutionary dynamics.

Interactions between virulent parasites and their hosts are frequently characterized by large fluctuations in host population size (e.g., Utida 1957; Pimentel 1968; Hudson et al. 1998; Bohannan and Lenski 2000). Such fluctuations can result in host genetic bottlenecks, which can theoretically have major consequences for host–parasite coevolutionary dynamics. First, bottlenecks can increase the likelihood of alleles going to fixation (Quigley et al. 2012; Gokhale et al. 2013), which may subsequently constrain coevolutionary dynamics. Second, they can reduce the relative amount of genetic variation in host versus parasite populations, which in turn may produce the parasite with an evolutionary advantage (Slatkin 1987; Gandon and Michalakis 2002; Morgan et al. 2005; Pal et al. 2007; Morran et al. 2011), manifesting as greater parasite infectivity to sympatric hosts and increased adaptation of parasites to local versus foreign hosts. Third, bottlenecks are likely to promote stochastic divergence between populations (i.e., genetic drift), further facilitating parasite adaptation to their local host populations (Gandon and Nuismer 2009).

Despite the theoretical importance of host genetic bottlenecks for host–parasite coevolution, we are unaware of any experimental tests. Here, we carry out such a test using coevolving populations of the bacterium *Pseudomonas fluorescens* (isolate SBW25) and its naturally associated bacteriophage ϕ2. In this system, coevolution is largely characterized by arms race dynamics with hosts becoming resistant to a wider range of parasite genotypes over time and vice versa (Buckling and Rainey 2002; Buckling et al. 2006; Poullain et al. 2008), and local adaptation can occur as a result of populations following divergent evolutionary trajectories (Buckling and Rainey 2002). We manipulated host population bottleneck size, to ask the following specific questions: (i) Do coevolutionary dynamics occur at a slower rate when hosts experience more severe size bottlenecks, as a result of their reduced evolutionary potential? (ii) Do more severe bottlenecks reduce sympatric host resistance (i.e., provide viruses with a relative advantage)? (iii) Do bottlenecks enhance local adaptation as a result of genetic drift or an increase in the adaptive potential of phages.

## Material and Methods

### COEVOLUTION EXPERIMENT

We imposed three different bottleneck sizes to obtain bacterial populations of putatively low, medium, and high diversity using the following procedure (note that diversity was affected in the expected way following these bottleneck events; see Results). For each treatment, 12 replicate microcosms, containing 6 ml of King's B (KB) Media, were inoculated with 10^8^ bacterial cells (derived from a single *P. fluorescens* SBW25 clone) and 10^5^ clonal phage particles (obtained from a single plaque of a clonal phage ϕ2). Bacteria and phages were then allowed to coevolve in static microcosms at 28°C for a 48 h period, before transferring a subset of bacterial clones, and their sympatric phages, to fresh microcosms for a total of eight transfers (∼56 bacterial generations). At each transfer we imposed a host population bottleneck by spotting serial diluted culture (100 μl of 10^−6^, 10^−4^ diluted and undiluted culture, corresponding to small (S), medium (M), and large (L) host populations, respectively) onto standard KB agar plates. After overnight incubation, clones were transferred to microcosms by streaking a pipette tip diagonally across the bacterial lawn. Bacterial populations were then grown overnight, before 1% of the total population was transferred into fresh microcosms, together with 10^5^ phage particles that had been isolated from the appropriate transfer by centrifuging cultures with 10% chloroform, which lyses and pellets bacterial debris (Buckling and Rainey 2002). Note that although the presence of phage in serial diluted culture resulted in selection for increased resistance during overnight incubation, the extent of this effect was independent of the imposed bottleneck size (see Supplementary Information). Every second transfer, samples (600 μl) of the total population were frozen at –80°C in 20% glycerol v:v KB solution.

### ASSAYS

We estimated phage densities through time from the number of plaque forming units (PFU) on lawns of exponentially growing ancestral SBW25 in 0.6% KB agar. In addition, resistance of bacterial populations against a given phage population was determined by streaking 20 independent bacterial clones across a line of (A): ancestral phage SBW25 ϕ2; (B) sympatric phage and (C) allopatric phage that had coevolved with bacteria from a different replicate of the same bottleneck treatment, resulting in six paired replicates in each treatment. For the most extreme bottleneck treatments, we also tested bacterial resistance against infectivity by allopatric phages isolated from the alternative treatment, yielding 12 paired replicates. To obtain a measure of overall resistance/infectivity in small versus larger host populations, we averaged across the different streak assays. Ancestral SBW25 was used on every plate as a control. A bacterial colony was defined as resistant if there was no growth inhibition; otherwise it was defined as sensitive.

Local adaptation was calculated for each pair of replicates as the mean difference in the proportion of local and foreign bacterial clones resistant to the two phage populations, removing any confounding effects of one phage or bacteria population having a higher mean infectivity or resistance than the other (Vos et al. 2009). When bacteria are locally adapted this measure is positive, when phages are locally adapted this measure is negative. Based on our survivorship assay, resistance was assessed for the second transfer only (see Results).

### STATISTICAL ANALYSES

For all analyses, we used the statistical package *R* Version 3.1.3 (R Development Core Team; http://www.r‐project.org). Since phages were rapidly driven extinct in some treatments, we used a Cox proportional hazards model to assess the effect of bottleneck size on phage survivorship over coevolutionary time. Proportional hazards assumptions of the Cox regression were examined using Schoenfeld residuals, after which post‐hoc contrasts were computed using the “nparcomp” package (Konietschke et al. 2014). To account for unequal between‐treatment variances, we used a generalized least squares (GLS) model with a constant variance function to test for the effect of host population bottleneck size on phage density after two transfers. The most parsimonious model was arrived at by comparing model fits using *F‐*tests of likelihood ratios. We used the same approach to test for the effect of bottleneck size on mean levels of infectivity and resistance in small versus large host populations. Additionally, we used a 1‐sample *t*‐test to assess whether local adaptation differed significantly from zero. Finally, we used Kruskal–Wallis tests to quantify the effect of bottleneck size on bacterial resistance to infection by sympatric and allopatric phages as well as the nature of local adaptation. In case of significant treatment effects, we used Dunn's tests with Bonferroni's adjustments to carry out post‐hoc comparisons.

## Results

### PHAGE PERSISTENCE

**Figure 1:**
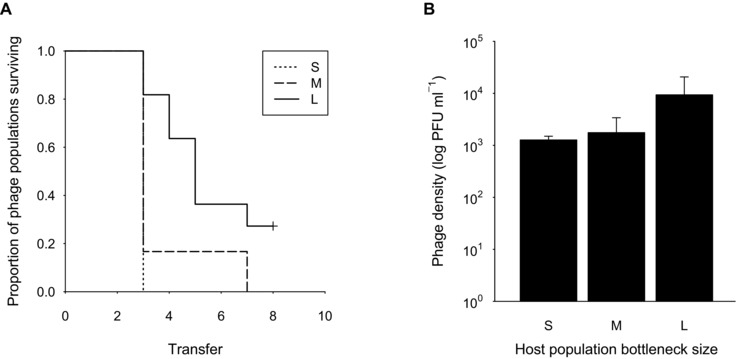
Proportion of phage populations surviving over coevolutionary time in hosts that had experienced large (bold, *n* = 12), medium (dashed, *n* = 12) or small (dotted, *n* = 12) population size bottlenecks (A). Density of phages after 13 generations of coevolution (transfer 2) in small (S), medium (M), and large (L) host populations (B). Bars display one SE.

We set out to measure coevolutionary dynamics, but to our surprise many phage populations rapidly reached undetectable levels in the majority of replicates after eight transfers (Fig. 1A). Specifically, mean time to extinction was significantly faster for phages coevolving with more severely bottlenecked hosts (*χ^2^_2, 34_*= 14.74, *P* < 0.001; mean ± SE transfers to extinction in S = 3.00 ± 0.00; M = 3.67 ± 0.45; L = 5.45 ± 0.59; post‐hoc tests S‐L: *z* = 3.56, *P* = 0.001; M‐L: *z* = 2.60, *P* = 0.03, and S‐M: *z* = 1.50, *P* = 0.29). After two transfers, phage densities were significantly lower compared to the inoculation density (one sample *t*‐test: *t* = 9.74, *P* < 0.001; Fig. 1B), and there was nonsignificant tendency for population size reductions to be greatest when host bottlenecks were most severe (GLS on log PFU: *F_2,33_* = 3.11, *P* = 0.06). Consistent with the theoretical expectation, increasing the severity of the host population bottleneck size did slow down coevolutionary dynamics (in that coevolution was stopped), but this was because of phage extinction as opposed to reduced host evolutionary potential.

### Bacterial resistance to phages

**Figure 2:**
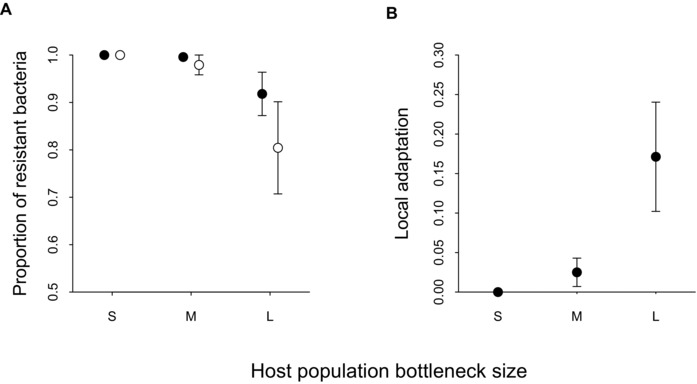
The effect of host population bottleneck size (S = small, M = medium, and L = large) on (A) bacterial resistance to sympatric (filled dots) and allopatric (open dots) phages; and (B) local adaptation measured after two transfers. Bars display one SE.

**Figure 3:**
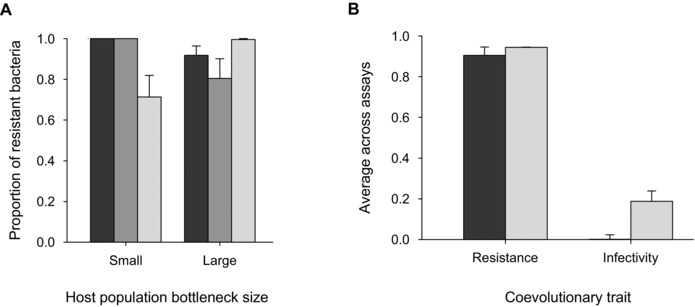
(A) Bacterial resistance to infection by phages (sympatric = black, within‐allopatric = dark gray, between‐allopatric = light gray) in small versus large host populations. (B) Bacterial resistance and phage infectivity in small (black) and large (gray) host populations when averaged across resistance assays. Bars display one SE.

Phages were detected in all populations after two transfers (Fig. 1B), so we measured bacterial resistance at this point. In line with previous work in this system, all isolated bacterial clones were resistant to infection by the ancestral phage, and all evolved phage populations were able to infect the ancestral bacterial strain. However, in contrast to our expectation, host resistance to infection by sympatric phages actually increased, rather than decreased, with decreasing host population size (*χ^2^_2, 34_*= 6.29, *P* = 0.04; Fig. 2A; post‐hoc tests S‐L: *z* = 2.38, *P* = 0.03; M‐L: *z* = 1.87, *P* = 0.09, and S‐M: *z* = –0.52, *P* = 0.90), which is consistent with our finding of reduced phage survivorship following severe bottleneck events. Similarly, allopatric phages were able to infect a larger proportion of within‐treatment bacterial clones as host population size increased (*χ^2^_2, 34_*= 8.21, *P* < 0.05; Fig. 2A; post‐hoc tests S‐L: *z* = 2.70, *P* = 0.01; M‐L: *z* = 2.18, *P* = 0.04, and S‐M: *z* = –0.52, *P* = 0.90). Conversely, bacterial resistance to infectivity by allopatric phages isolated from the alternative bottleneck treatment was significantly lower in the smallest host populations (*χ^2^_2, 34_*= 7.39, *P* < 0.01; Fig. 3A). Finally, although mean levels of resistance did not vary as a function of host population bottleneck size (GLS on mean resistance: *F*_1, 70_ = 0.72, *P* = 0.40; Fig. 3B), mean phage infectivity was significantly lower in severely bottlenecked hosts (GLS on mean infectivity: *F_1, 70_* = 13.49, *P* < 0.001).

### Local adaptation

We anticipated that the combination of host bottlenecks providing phage an evolutionary advantage and the increase in stochastic divergence between populations would increase the likelihood of parasite local adaption. Instead, but consistent with our other results, bacteria were locally adapted to phages (Fig. 2B; one sample *t*‐test: *t* = 2.48, *P* = 0.02). Moreover, the magnitude of bacterial local adaption depended on population bottleneck size (*χ^2^_2, 34_*= 13.79, *P* < 0.01; Fig. 2B), increasing with size (post‐hoc tests S‐L: *z* = 3.56, *P* < 0.01; M‐L: *z* = 2.70, *P* = 0.01, and S‐M: *z* = 0.86, *P* = 0.59).

## Discussion

Host–parasite interactions are often characterized by large fluctuations in host population size, and we investigated how such host bottlenecks affected coevolution between a bacterium and a virus. Theoretical studies have demonstrated that bottleneck events can affect coevolutionary dynamics by: (i) increasing the likelihood of allele fixation (Quigley et al. 2012; Gokhale et al. 2013) and constraining coevolutionary dynamics (ii) reducing the evolutionary potential of hosts versus parasites (Gandon and Michalakis 2002) and (iii) promoting the emergence of local adaptation when genetic drift is not too strong (Gandon and Nuismer 2009). While bottlenecks constrained coevolution, this was because resistance alleles, which phages were apparently unable to overcome, went to fixation, resulting in phage extinction. Moreover, this fixation of resistance alleles reduced parasite local adaptation. Together these results indicate that, in contrast to our predictions, bottlenecks provide bacterial populations with an evolutionary advantage against their viruses, and reduce local adaptation.

Why were phages driven to extinction during the early stages of coevolution with hosts of reduced diversity? In this and many other bacterium‐phage systems, bacteria are found to be ahead in the coevolutionary arms race and typically display high levels of sympatric resistance, with relatively few susceptible clones at any time (Buckling and Brockhurst 2012). However, susceptible clones are likely to be crucial for coevolution, since phages rely on these clones to grow and persist. Indeed, previous studies have shown that the loss of sensitive clones can result in phage extinction (Schrag and Mittler 1996; Lythgoe and Chao 2003; Brockhurst et al. 2005, 2006). In our experiment, sensitive clones were likely lost by chance in bottlenecked populations, as apparent from the increased levels of sympatric resistance following strong bottleneck events. In the absence of susceptible hosts, phage populations were presumably unable to achieve sufficiently high population sizes to generate novel infectivity mutations and persist. Importantly, our results therefore indicate that sensitive bacteria are not only important for maintaining coexistence between hosts and parasites in the absence of parasite infectivity evolution (Schrag and Mittler 1996; Lythgoe and Chao 2003; Brockhurst et al. 2005, 2006), but also play a crucial role in coexistence during coevolutionary interactions.

Note that phages were not removed prior to imposing the different size bottlenecks, because it is very hard to do so without causing some collateral damage to bacterial cells. The presence of phage could potentially have resulted in continued selection for host resistance during overnight incubation, thereby expediting phage extinction. The key issue is whether this selection differs between bottleneck treatments. We therefore explicitly assayed how the bottleneck procedure affected bacterial resistance (see Supplementary Information). Our results demonstrate that resistance to infection by ancestral phage did increase as a likely result of continued selection against sensitive bacterial cells, but crucially the extent of this effect did not differ between bottleneck treatments (Fig. S1), and hence cannot explain our results.

An additional explanation for our observation of phage extinction is a reduction in phage infectivity evolution with more severe host bottlenecks. It has been previously reported that phage infectivity quickly deteriorates when subjected to strong repeated size bottlenecks (Chao 1990). In our experiment, phage densities steadily decreased during the early stages of coevolution. However, phage population sizes prior to extinction were still typically in excess of 10^3^ in the severe host bottleneck treatment, suggesting phage bottlenecks were probably not responsible for the observed reduction in infectivity. However, there was a tendency for phage population sizes to be lower in the severe bottleneck treatment, which likely reduced the potential for infectivity evolution to occur. In other words, the extinction of phages with severe host bottlenecks probably reflects eco‐evolutionary feedbacks between high levels of resistance and resultant reductions in phage population size constraining subsequent phage infectivity evolution.

Theoretical studies of coevolutionary dynamics have demonstrated that strong genetic drift may prevent the emergence of local adaptation depending on the underlying specificity of the host–parasite interaction (e.g., Gandon and Nuismer 2009). Our finding of reduced local adaptation with increasing bottleneck severity can simply be explained by the fixation of resistance alleles in all populations. Crucially, these resistance alleles conferred resistance to sympatric and allopatric populations (as is typical of arms race dynamics, where resistance and infectivity increase in parallel between populations through time). Hence, rather than increasing phenotypic divergence between populations, divergence was actually decreased following repeated bottleneck events. Note that where we did observe local adaptation in the less severely bottlenecked populations, it was bacteria that were locally adapted, as has been observed in previous studies in this systems (Morgan et al. 2005).

These results suggest complex, but likely predictable, effects of host population bottlenecks on host–parasite coevolution. Where hosts are ahead in the arms race, as found here, bottlenecking is likely to be bad for local parasite persistence because of the stochastic loss of sensitive hosts in combination with selection for reduced phage infectivity. By contrast, where parasites are ahead, and there is not the reliance on sensitive hosts, host bottlenecking is likely to be beneficial to parasites, and the predictions outlined at the start are likely to apply. Indeed, this is why monocultures (a human‐imposed bottleneck) in the context of agriculture are typically viewed as bad for hosts, because parasites often evolve to overcome the one host genotype (King and Lively 2012). More generally, the results emphasize the importance of feedbacks between ecological and coevolutionary dynamics, and how these can qualitatively alter coevolutionary dynamics.

## DATA ARCHIVING

Dryad: doi.org/10.5061/dryad.9rr6v.

## Supporting information


**Figure S1**. The proportion of bacterial clones resistant to infection by ancestral phage before (black bars) and after imposing a host population size bottleneck (white and grey bars denote small and large host populations, respectively).
