# Supplementary material for: Host population bottlenecks drive parasite extinction during antagonistic coevolution
Source: Evolution. 2015 Dec 30;70(1):235–40. doi: 10.1111/evo.12837 (PMC4736460; doi:10.1111/evo.12837)
Supplement: Supplementary file 1 — Figure S1. The proportion of bacterial clones resistant to infection by ancestral phage before (black bars) and after imposing a host population size bottleneck (white and grey bars denote small and large host populations, respectively). [file EVO-70-235-s001.docx]

SUPPORTING INFORMATION

Additional Supporting Information may be found in the online version of this article:

**Figure S1:** The proportion of bacterial clones resistant to infection by ancestral phage before (black bars) and after imposing a host population size bottleneck (white and grey bars denote small and large host populations, respectively). In short, six replicate KB microcosms were inoculated with 10^8^ bacterial cells (derived from a single *P. fluorescens* SBW25 clone) and 10^5^ clonal phage particles (obtained from a single plaque of a clonal phage φ2). Bacteria and phages were then allowed to coevolve statically at 28° C for a 48h period, after which we isolated 24 individual bacterial clones per replicate (*before* treatment, *n* = 6). We subsequently imposed a bottleneck by spotting serial diluted culture onto KB agar plates (i.e., 100 μl of 10^-6^ and undiluted culture, corresponding to small and large host populations, respectively). After overnight incubation, clones were transferred to microcosms by streaking a pipette tip diagonally across the bacterial lawn. We isolated 24 bacterial clones per replicate (*after* treatment, *n* = 6 for small and large host populations) and determined the resistance of all isolated clones to infection by ancestral phage using a streak assay. Crucially, results from a Kruskal-Wallis test demonstrate that the observed increase in bacterial resistance following a bottleneck event was independent of its size (*χ^2^_2_* = 3.81, *p* = 0.15).

**Figure S1.**
